# Improved anchoring nails: design and analysis of resistance ability: Tensile test and finite element analysis (FEA) of improved anchoring nails used in temporomandibular joint (TMJ) disc anchor

**DOI:** 10.1186/s12903-018-0606-3

**Published:** 2018-08-25

**Authors:** Z. H. Zhou, X. Z. Chen, X. W. Chen, Y. X. Wang, S. Y. Zhang, S. F. Sun, J. Z. Zhen

**Affiliations:** 1 Department of Oral and Maxillofacial Surgery, School of Medicine, Ninth People’s Hospital, Shanghai Jiao Tong University, 639 Zhi Zao Ju Road, Shanghai, 200011 People’s Republic of China; 2 Department of Stomatology, Tongren Hospital Affiliated to Shanghai Jiao Tong University School of Medicine, Shanghai, 200336 People’s Republic of China; 3 Department of Oral Surgery, Shanghai Key Laboratory of Stomatology & Shanghai Research Institute of Stomatology, College of Stomatology, Ninth People’s Hospital, Shanghai Jiao Tong University School of Medicine, No. 639, Zhi-Zao-Ju Road, 200011 Shanghai, People’s Republic of China

**Keywords:** Temporalmandibular joint disc anchorage, Mandibular condyle, Anchoring nail, Suture, Tensile test, Finite element analysis, FEA

## Abstract

**Background:**

Anchorage is one of the most important treatments for severe temporomandibular joint disorder (TMD). Anchoring nails have shown great success in clinical trials; however, they can break under pressure and are difficult to remove. In this study, we aimed to evaluate an improved anchoring nail and its mechanical stability.

**Methods:**

The experiment consisted of two parts: a tensile test and finite element analysis (FEA). First, traditional and improved anchoring nails were implanted into the condylar cortical bone and their tensile strength was measured using a tension meter. Second, a three-dimensional finite element model of the condyles with implants was established and FEA was performed with forces from three different directions.

**Results:**

The FEA results showed that the total force of the traditional and improved anchoring nails is 48.2 N and 200 N, respectively. The mean (±s.d.) maximum tensile strength of the traditional anchoring nail with a 3–0 suture was 27.53 ± 5.47 N. For the improved anchoring nail with a 3–0 suture it was 25.89 ± 2.64 N and with a 2–0 suture it was above 50 N. The tensile strengths of the traditional and improved anchoring nails with a 3–0 suture was significantly different (*P* = 0.033–< 0.05). Furthermore, the difference between the traditional anchoring nail with a 3–0 suture and the improved anchoring nail with a 2–0 suture was also significantly different (*P* = 0.000–< 0.01).

**Conclusion:**

The improved anchoring nail, especially when combined with a 2–0 suture, showed better resistance ability compared with the traditional anchoring nail.

## Background

Temporomandibular joint disorder (TMD) is a common condition, with an approximate prevalence ranging from 13 to 87% [1]. Considering the limitations of non-surgical treatments, including medications [2], splints [3], physical therapy [4], and trigger point injections [5], surgical intervention is needed in severe cases of TMD [6, 7].

Previous clinical reports reveal that the results of surgical temporomandibular joint (TMJ) disc repositioning procedures have been variable due to long-term instability [8]. In 2001, an open joint procedure using Mitek anchoring nails (Mitek mini anchor, Mitek Products Inc., Westwood, Mass) showed great success in both clinical trials and radiography [9]. Despite their success, MiTek anchoring nails still have the following problems: (1) once fixed in the cortical bone, compared with other types of Mitek anchor, Mitek mini anchoring nails tend to break easily under pressure; (2) once the wings are twisted, extraction of the anchoring nail becomes difficult. Spallaccia et al. [10] (2013) described an anchorage surgery using bioabsorbable microanchor nails. Postoperative MRI showed a low reposition rate (65.7% in 35 patients). To improve the success rate, reposition stability and implant safety, He et al. [11] (2015) applied Chinese-made anchoring nails in modified disc anchorage surgery. As the shape of the anchoring nail does not fit perfectly with the anatomical structure of a condyle, 7.47% of patients experienced postoperative friction in the parotideomasseteric region [12]. Our group has previously modified the traditional Chinese-made anchoring nail to reduce discomfort and improve stability. The anchoring nail is designed to be fully threaded, totally implanted in the cortical bone, and fixed with a 2–0 suture. However, the properties and safety of the improved anchoring nail have not yet been studied.

Therefore, in this study, we aimed to assess the mechanical performance of the improved anchoring nail compared with the traditional anchoring nail. In previous studies, tensile tests have been used to estimate the resistance ability of Mitek anchors [9]. With advances in computer science, finite element analysis (FEA) has become a useful tool that addresses the limitations associated with the TMJ structure and has tremendous advantages in many aspects [13, 14]. FEA is capable of modeling and analyzing shape, structure, and resistance ability and has become the most popular numerical theoretical method for TMJ biomechanics analysis. In our study, we used tensile tests and FEA to estimate the resistance ability of the improved anchoring nail. The hypothesis of this study was that the improved anchoring nail would show greater tensile strength compared with the traditional anchoring nail.

## Methods

### Tensile test

#### Subjects

From April 2015 to June 2016, 10 patients (4 males and 6 females, aged 20–72 years old) undergoing TMJ replacement at the Department of Oral Surgery, the Ninth People’s Hospital Affiliated to Shanghai Jiaotong University School of Medicine were selected. Condyle specimens were collected, wrapped with wool yarn immersed in normal saline, and preserved at − 20 °C. This study was approved by the Ethics Committee of Shanghai JiaoTong University School of Medicine.

#### Anchoring nails

**Fig. 1:**
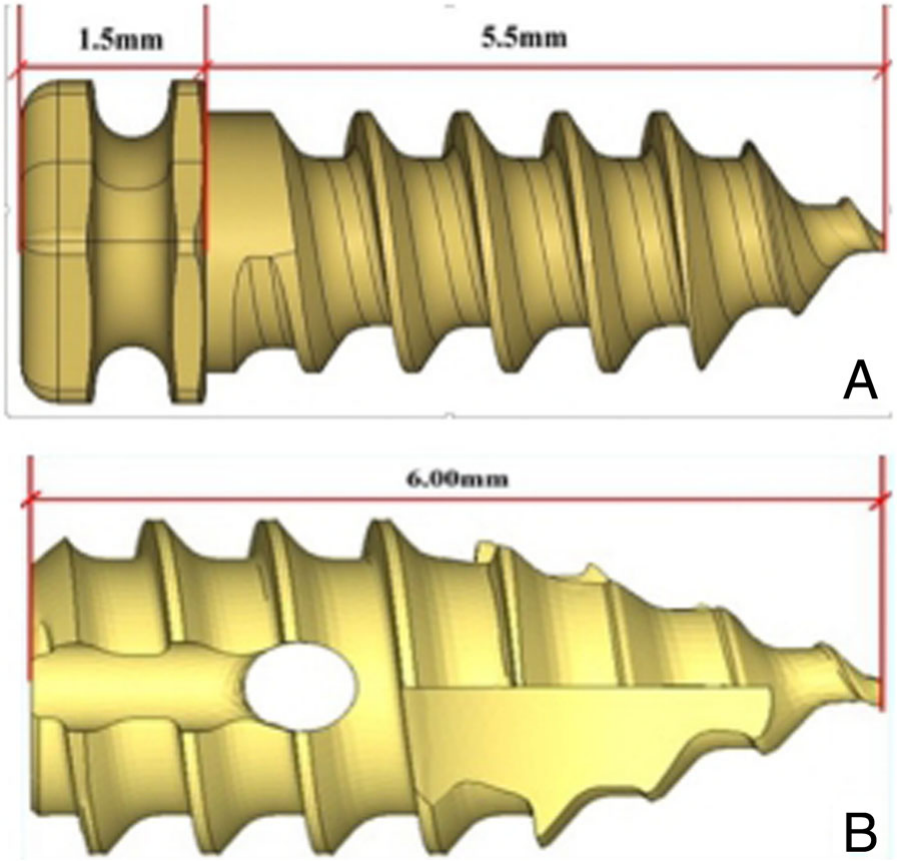
The two types of anchoring nails. **a** Traditional anchoring nail. **b** Improved anchoring nail

Both traditional and improved anchoring nails (Cixi City Cibei Dental Instrument Co., Ltd., Cixi, Zhejiang, China) were made of titanium alloy. The total length of the traditional anchoring nail was 7 mm, with 1.5 mm nut thicknesses, 5.5 mm thread length, 2.8 mm nut diameters, and 2.0 mm thread diameters. The transition between the head and thread was smooth and a groove was designed for the knotting of the suture, which can only be fixed with a 3–0 suture (Fig. 1a). The improved anchoring nail had a length of 6 mm and a diameter of 3.0 mm. There was a small hole in the upper-middle part of the anchoring nail, with two grooves connected to the head. The upper part of the grooves was smooth for the placing and knotting of the suture (Fig. 1b).

#### Sutures

In this study, 3–0 and 2–0 nylon sutures (Ethibond *Excel, Green Braided Polyester Suture, Ethicon, Inc.) were used. The sutures were 90 cm in length with one suture needle at each end. Each suture was divided into two in the middle.

#### Implantation procedure

**Fig. 2:**
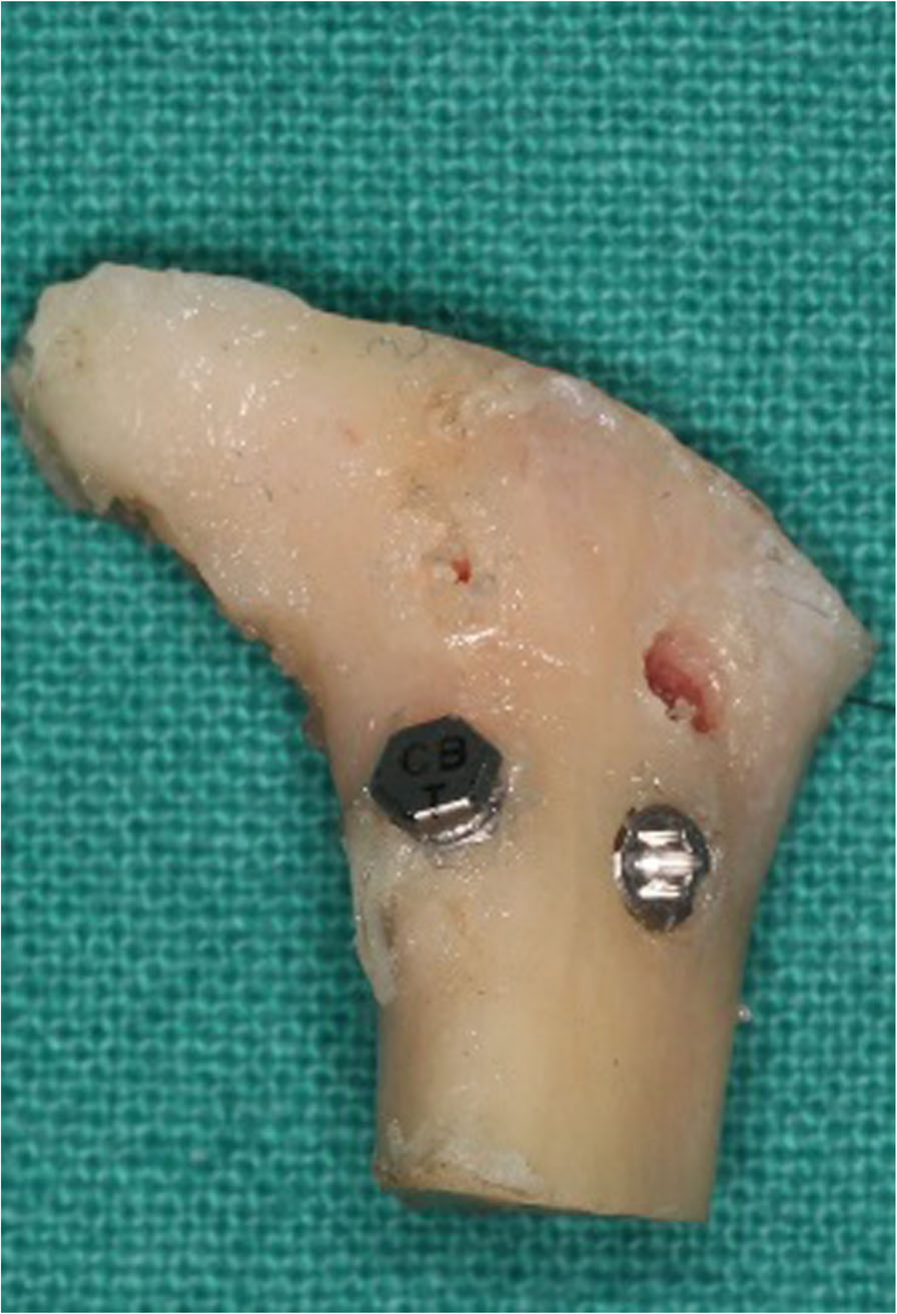
Condyle specimen. Traditional and modified anchoring nails were implanted in the condyle

Traditional and improved anchoring nails were implanted 10–15 mm below the inferior margin of the posterior inclined plane of the condylar process. The two anchoring nails were placed symmetrically and the distance between them was more than 3 mm (Fig. 2).

#### Tensile test

A tension meter ((Cixi City Cibei Dental Instrument Co., Ltd., Cixi, Zhejiang, China) was used for tensile tests. In the lower part, specimens were immobilized with steel wires to a clamping board. In the upper part, the suture was directly immobilized to the clamping board.

#### Statistical analysis

SPSS 17.0 software (Chicago, IL, USA) was used for statistical analysis. The maximum bearable tension forces of the sutures were analyzed by descriptive statistics and reported as *x ± s. d*. The difference in tension readings between the two sutures used for the traditional anchoring nail was compared using a *t*-test. (The traditional anchoring nail does not match a 2–0 suture.) The difference in tension readings between the two sutures used for the improved anchoring nail was compared using a Kruskal-Wallis test. *P* < 0.05 was considered significant.

### Finite element analysis

#### FEA tool

We used three-dimensional modeling software (Hypermesh, Altair Engineering Inc.) and analysis programs (LS-DYNA, LSTC Inc.) to regulate the network structure and make it more homogeneous, create the solid model, and conduct the stress analysis using the finite element procedure.

#### Finite element model

**Fig. 3:**
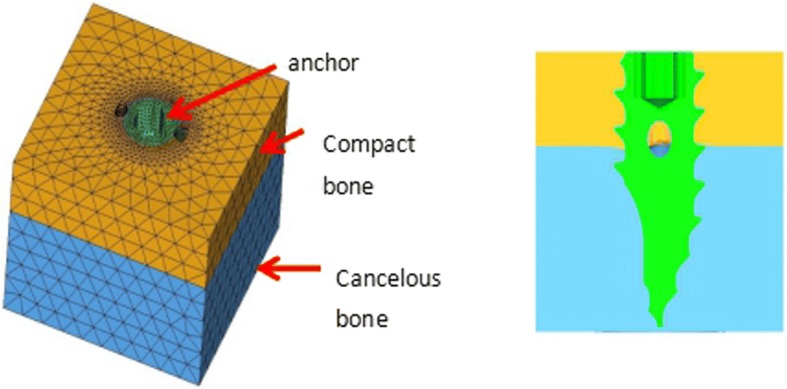
Finite element computer-aided design model

A three-dimensional computer-aided design model of the anchoring nails was established and used as the mesh model for the FEA. The finite element model consisted of a first-order tetrahedral mesh, a total of 139,000 units, and 29,000 nodes. (Fig. 3).

#### Data processing

The FEA results are a stress result accumulated gradually by deformation. Consequently, FEA transforms an engineering stress–strain curve to a true stress–strain curve. The formulae for the transformation are as follows:

- True stress = (1 + engineering strain) × engineering stress.
- True strain = ln(1 + engineering strain).

#### Process

**Fig. 4:**
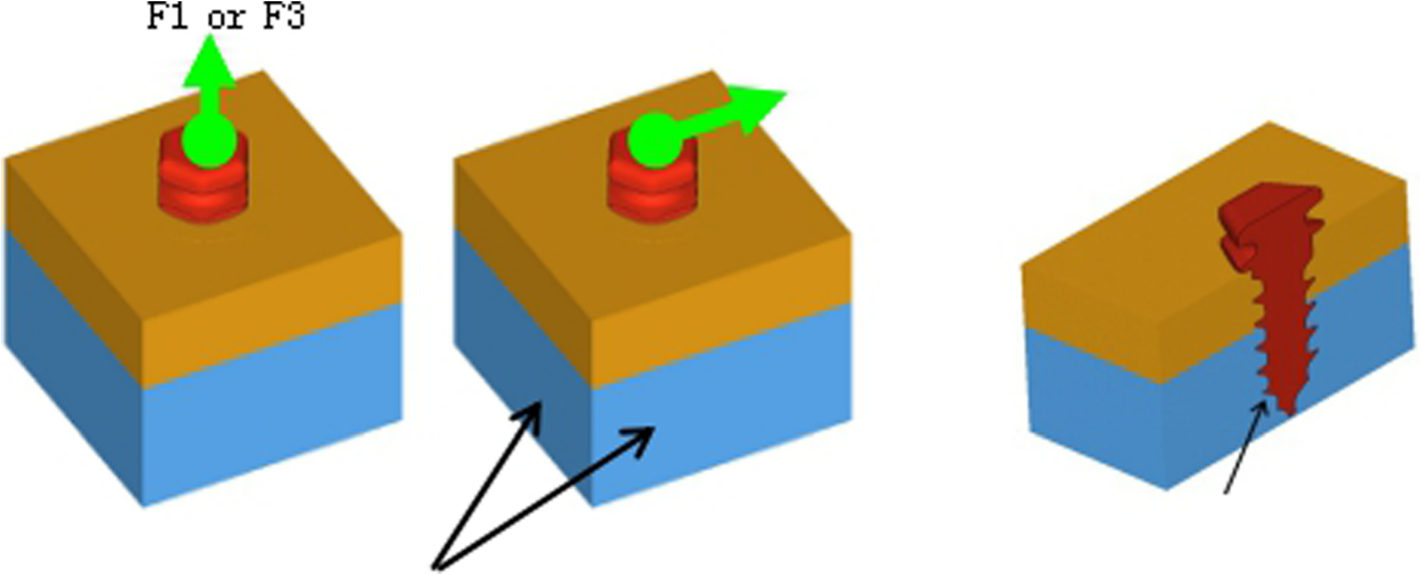
Three types of force

The directions of force, including vertical, level, and vertical rotation forces, were selected according to previous studies on TMJ disk movement [15–17]. The main stress point of the anchoring nail was analyzed by FEM.(Fig. 4).

## Results

Neither fracture of the cortical bone nor fracture or loosening of the anchoring nails occurred during the implantation, and the anchoring nails were all successfully implanted into the cortical bone of the condylar process. Only sutures were damaged during the tensile tests, and neither the anchoring nails nor the cortical bone were damaged. As the 2–0 suture used for the improved anchoring nail was still not fractured at the maximum tension reading on the tension meter of 50 N, the tensile strength was recorded as above 50 N (Table 1).

**Table 1:** Mean maximum bearable tension forces of sutures under different conditions (*N*, x ± s)

Suture	Number of measurements	Mean maximum bearable tension force (F/N)	Range (F/N)
3–0 suture	20	28.74 ± 3.52	21.15~ 34.17
Conventional anchor nail (with 3–0 suture)	20	27.53 ± 5.47	17.84~ 37.80
Improved anchor nail (with 3–0 suture)	20	25.89 ± 2.64	21.32~ 33.83
Improved anchor nail (with 2–0 suture)	20	50	≥50
2–0 suture	20	50	≥50

**Fig. 5:**
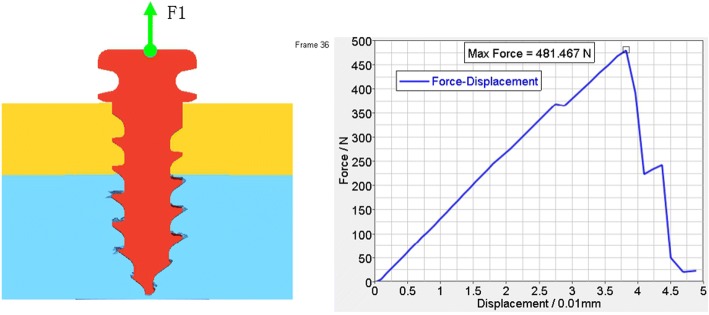
The total vertical force of the traditional anchoring nail is 481.467 N

**Fig. 6:**
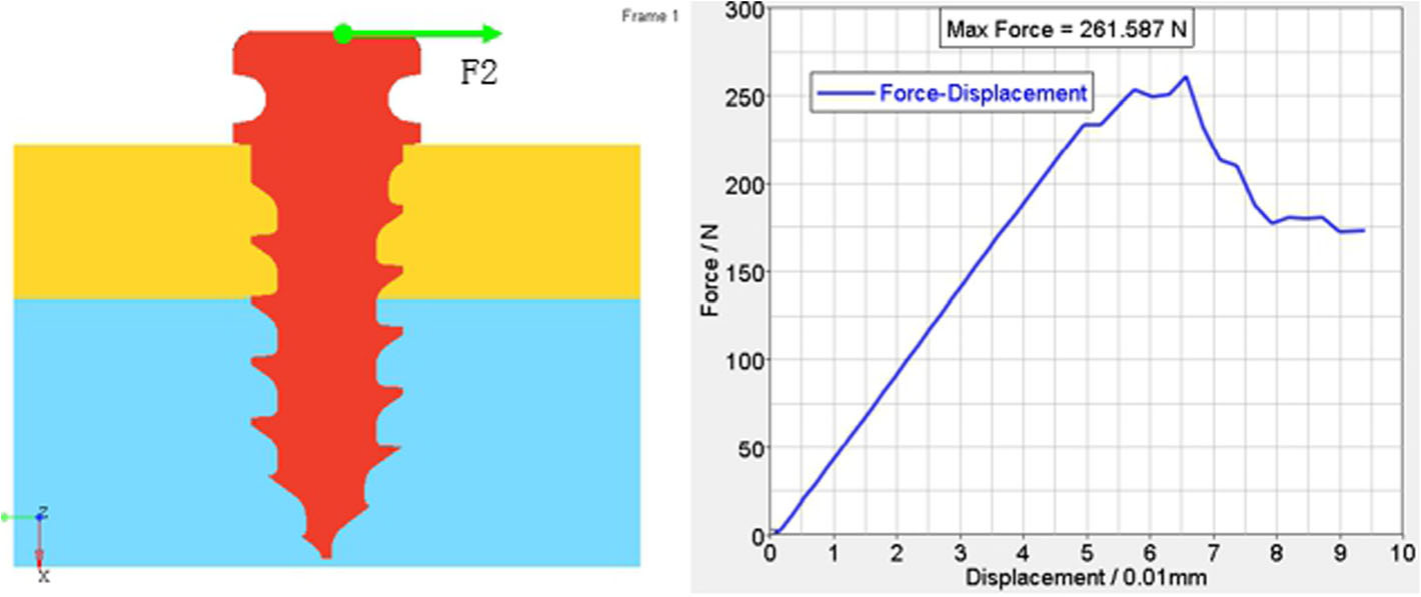
The total level force of the traditional anchoring nail is 261.6 N

**Fig. 7:**
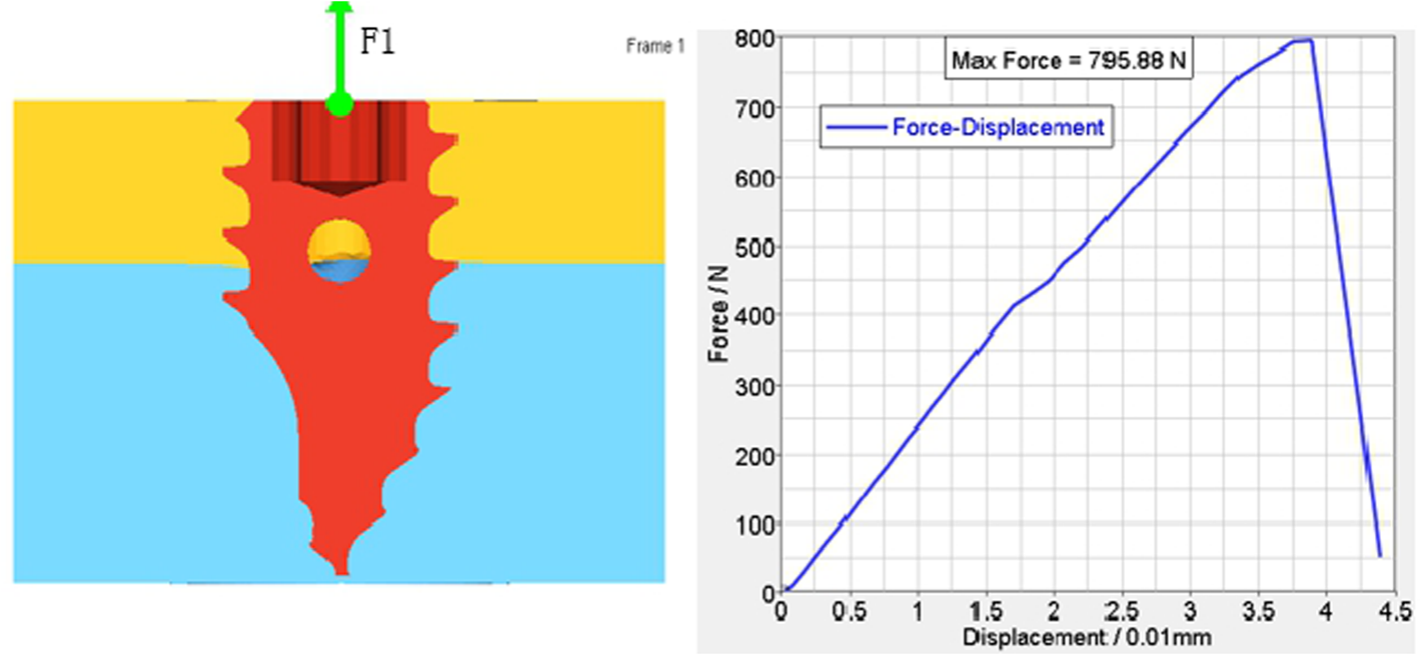
The total vertical force of the improved anchoring nail is 795.88 N

**Fig. 8:**
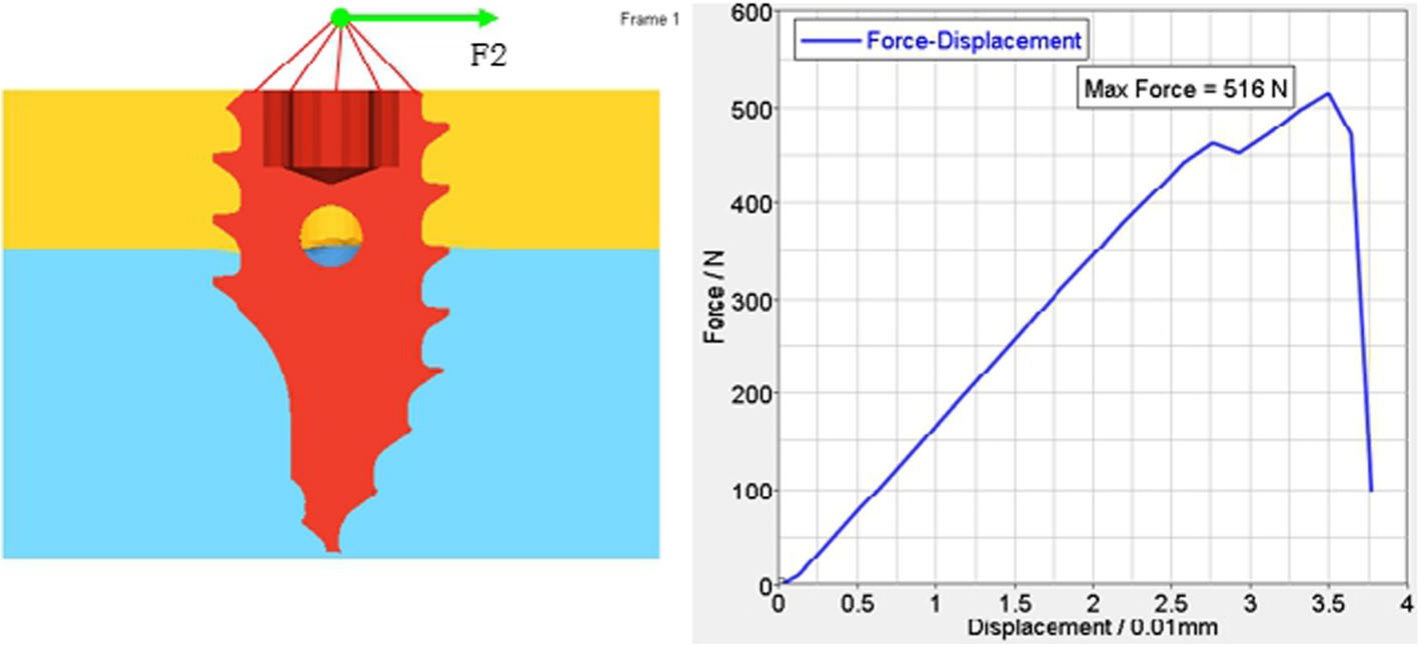
The total vertical force of the improved anchoring nail is 516 N

**Fig. 9:**
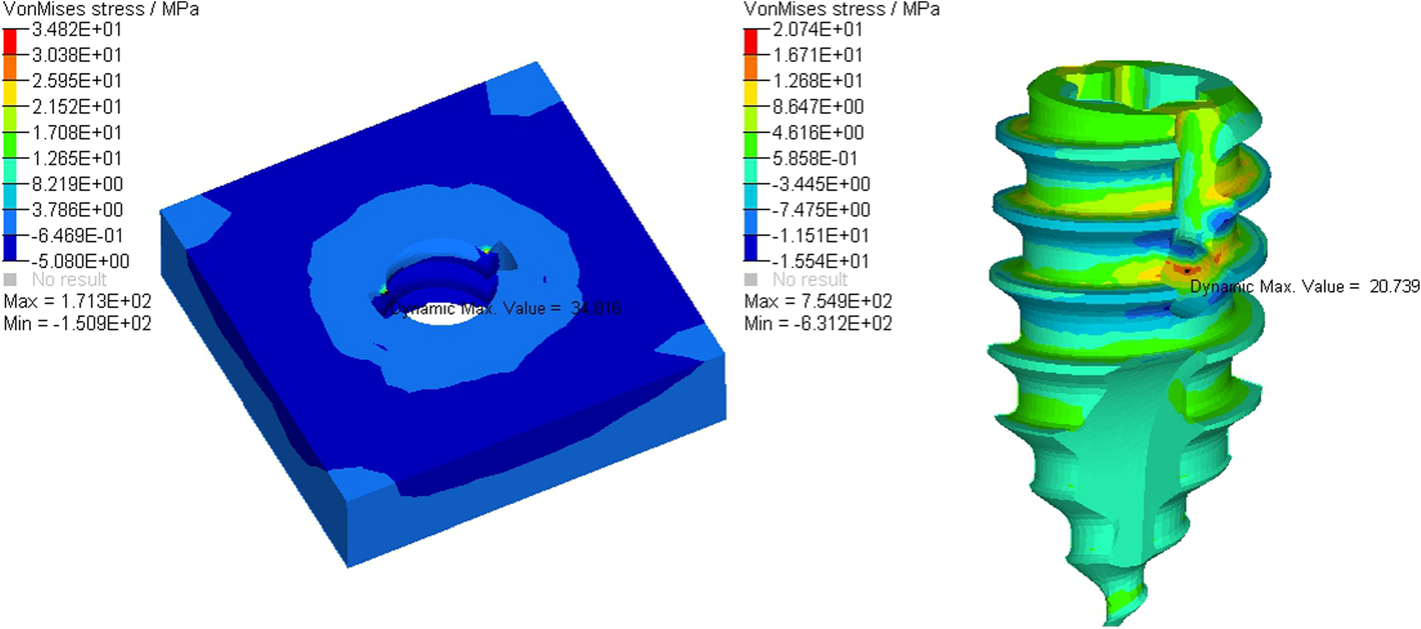
The stress distribution between the cortical bone and the anchoring nail

According to the FEA of the traditional anchoring nail in condyle, the total vertical force is 481.467 N (Fig. 5), the total level force is 261.587 N (Fig. 6), and the total vertical rotation force is 48.2 N. Therefore, the total force of the traditional nail is 48.2 N. For the improved anchoring nail, the total vertical force is 795.88 N (Fig. 7), the total vertical force is 516 N (Fig. 8) and the total vertical rotation force is 200 N. Therefore, the total force of the improved nail is 200 N, which is twice that of the traditional anchoring nail. In the FEA of the different anchoring nail, regardless of the direction the force, the main force points are in the condylar cortical bone rather than the cancellous bone (Fig. 9).

## Discussion

Common applications of anchoring nails include repair of the medial canthal ligament, muscle reattachment, TMJ disc repositioning, and other craniofacial surgery [17–19]. Mehra and Wolford [9](2001) reported the first case of using MiTek anchoring nails to reposition the TMJ disc, which achieved good clinical outcomes. However, the specimens they used were from non-living patients. Therefore, the bones had a lower bone density, higher brittleness, and higher risk of fractures, compared with bones taken from living patients, which was the main reason for the frequent bone damage observed. In the tensile test, we performed a control study by implanting the different types of nails in the same fresh specimen to exclude the effect of individual variations and to mimic the real clinical situation. The force applied in the tensile tests was parallel to the long axis of the anchoring nail and the minimum pull-out force was above 50 N. The improved anchoring nail was superior to the traditional anchoring nail.

Both of the anchoring nails used by Mehra and Wolford were implanted into the cortical bone after using a special puncher. If rejection occurs or the anchoring nails are deformed, damaged or misplaced, it is very difficult and traumatic to remove them. Furthermore, a few patients using traditional anchoring nails complained of discomfort in the anterior wall of the external auditory canal, which may be related to the protrusion of the anchoring nail nut. We modified the design of the anchoring nail based on MiTek anchoring nails and the anchoring nails used by He et al. [11](2015). The improved anchoring nail is much easier to implant and extract.

Tensile tests were conducted for the traditional and improved anchoring nails. It was found that the maximum tensile strength of the sutures used in the different anchoring nails varied. For the 3–0 suture, the *t*-test indicated a significant difference in tensile strength between the improved and traditional anchoring nails (*P* = 0.033–< 0.05). The tensile strength of the conventional anchoring nail with a 3–0 suture was higher than that of the improved anchoring nail with a 3–0 suture. The Kruskal-Wallis test showed that the difference was significant between the 3–0 and 2–0 sutures (*P* = 0.000–< 0.1). Therefore, the improved anchoring nail with a 2–0 suture was superior to the traditional and improved anchoring nails with a 3–0 suture (Table 2). After implantation, the tensile strength of the 3–0 suture did not vary considerably between the different anchoring nails, and the maximum tensile strength of the sutures in the different anchoring nails was generally smaller than the modulus of elasticity of the sutures. The method of tying sutures to the anchoring nails had little impact on the tensile strength of the sutures.

**Table 2:** Comparison of tension forces between traditional and improved anchoring nails

Group	Conventional anchor nail (3–0)/improved anchor nail (3–0)	Improved anchor nail (2–0)/conventional anchor nail (3–0)
F (N)	27.53 ± 5.47/25.89 ± 2.64	50/27.53 ± 5.47
*P* value	0.033	0.000

Compared with larger-suture anchoring nails used in plastic surgery [19] the two anchoring nails in our study had a lower retention force. Other studies have generally been conducted in swine thighbones or in other places in the human body where the bone density is higher. The cortical bone is thicker and the contact area of the anchoring nails was greater. Our measurements indicated that the retention force of the improved anchoring nails was above 50 N, which is higher than the lowest value reported in the above studies. Moreover, the length of the improved anchoring nail embedded in the cortical bone and the thread diameter was larger compared with the traditional anchoring nail.

In the analysis of the finite element model of the two types of anchoring nails, the force and form are given the same analysis. Moreover, we avoided the influence of condylar origin. When the vertical force is applied, the longitudinal pulling out force is often relatively larger compared with the considerable frictional force, due to the limited axial rotation of the anchoring nail. As for the horizontal force, an inversely proportional relationship existed between the lateral force size and force arm L. As the anchoring nail is an asymmetric structure, the lateral pull-out force would be slightly different following the change of lateral forces. When the vertical pull-out force is applied, it is assumed that the coefficient of friction between the anchoring nail and the bone is close to infinity. Furthermore, as both the anchoring nail and the bone have small coefficients of friction, the anchoring nail is mainly planted in the weaker intrinsic bone, which can barely resist anchoring nail rotation. Hence, a small longitudinal tension can make the anchoring nail come out. In the experiment, we used two types of anchoring nail under identical conditions to effectively simulate the actual situation of anchoring nails under stress.

Despite the advantages of this study, there are still some disadvantages. The sample size is small and we aim to enlarge the sample size to obtain more precise results. Moreover, we only performed this study in vitro. In this regard, we aim to verify the effectiveness in vivo and, eventually, in a clinical study.

## Conclusion

Both traditional and improved anchoring nails can be successfully implanted into the condyle without fracture of the anchoring nail or destruction of the cortical bone. The improved anchoring nail can resist a stronger pulling-out force compared with the traditional anchoring nail. It can be fixed with 2–0 suture, which substantially improves its resistance ability. It is also more convenient compared with a Mitek anchoring nail in terms of the implant and extraction processes [9].By conducting tensile tests and FEA of the two anchoring nails in the mandibular condyle, we conclude that the improved anchoring nail has better resistance ability compared with the traditional anchoring nail. The improved anchoring nail has the potential for clinical application; however, further research in animals and clinical experience is required.

## Abbreviations

FEA: Finite element analysis
FEM: Finite element analysis
TMD: Temporomandibular joint disorder
TMJ: Temporomandibular joint
